# Supplementary figures and images for: Nasopharyngeal Microbial Communities of Patients Infected With SARS-CoV-2 That Developed COVID-19
Source: Front Microbiol. 2021 Mar 17;12:637430. doi: 10.3389/fmicb.2021.637430 (PMC8010661; doi:10.3389/fmicb.2021.637430)

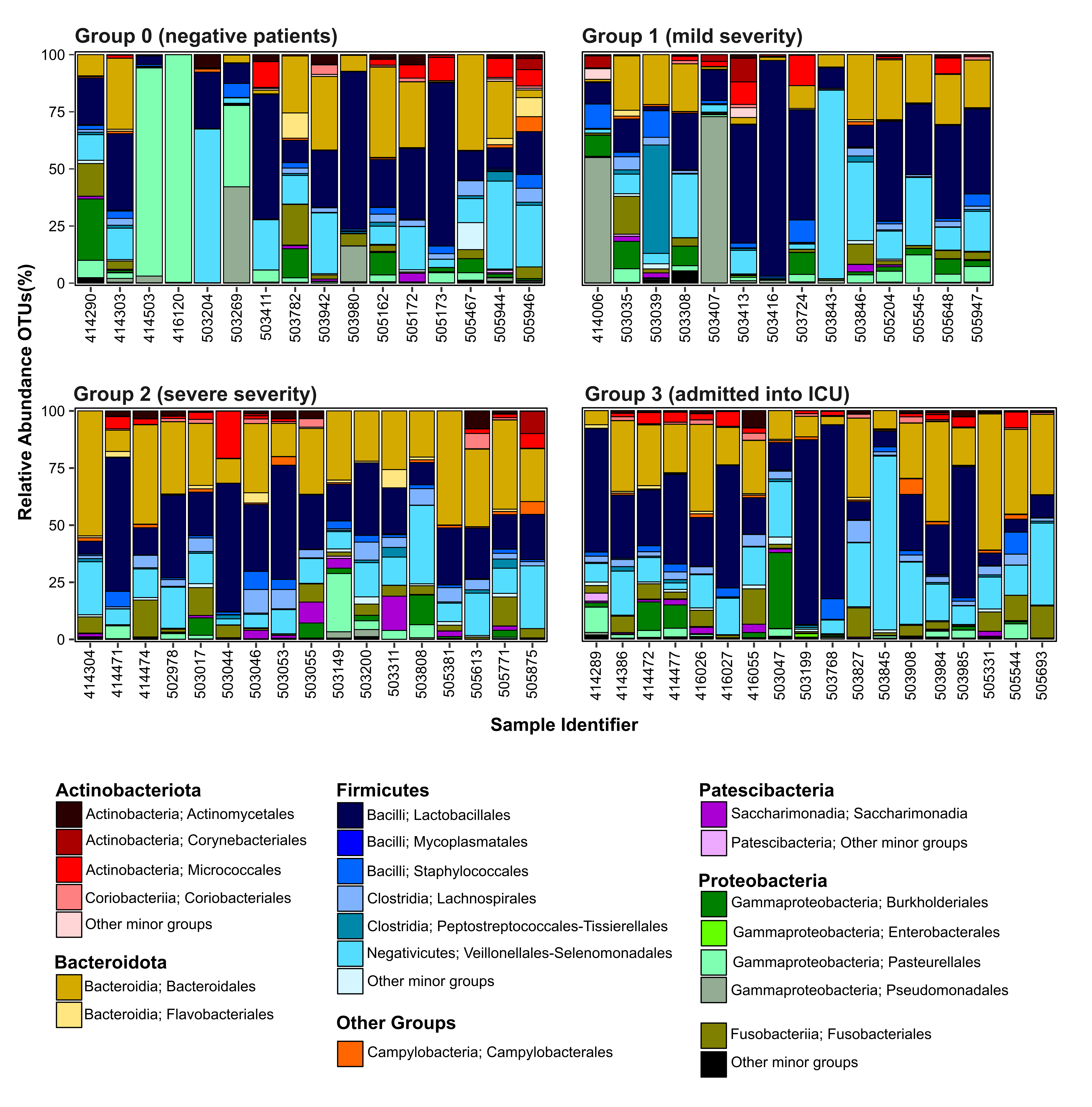

Supplement: Supplementary Figure 1 — Relative abundance of bacterial populations, at genus level, in the microbiome of patients within COVID-19 severity groups. Only microorganisms with a relative abundance greater than 0.5% are shown in the legend. [file Image_1.tif]

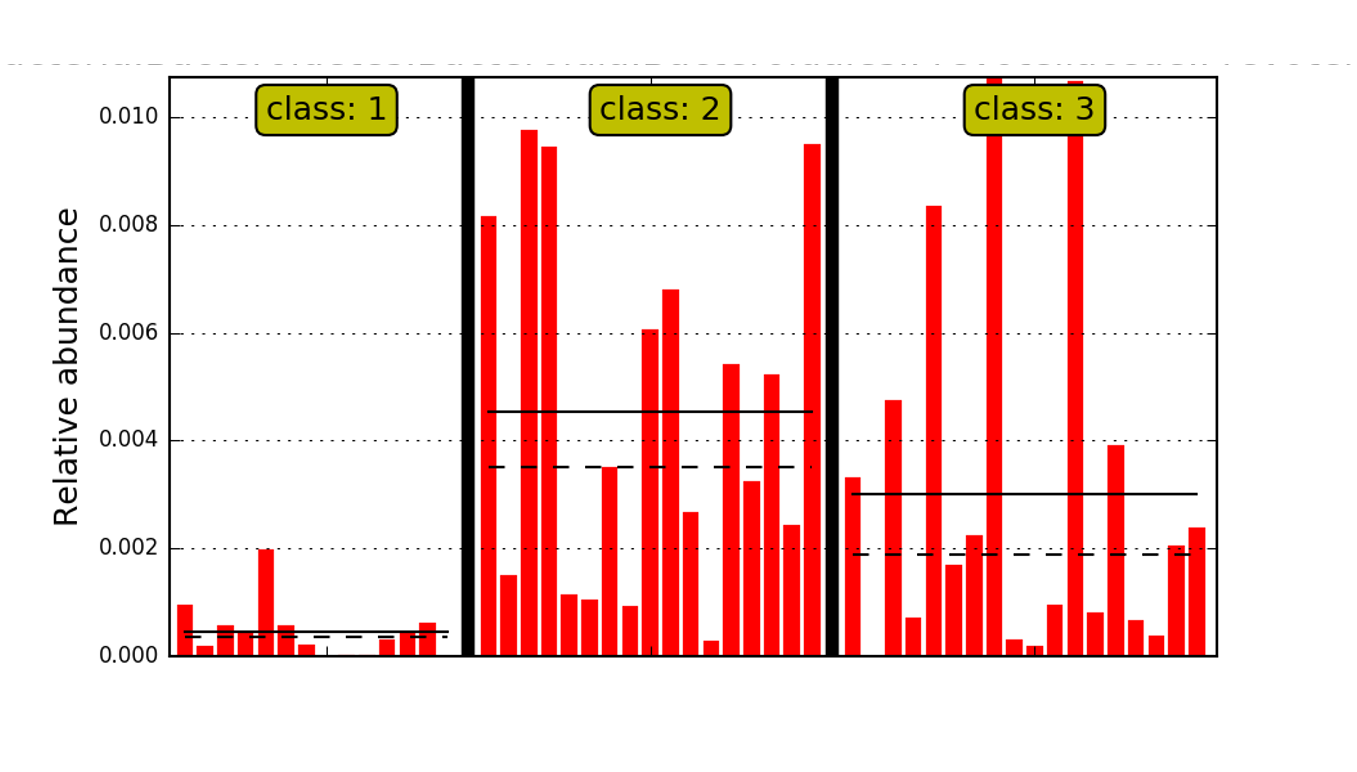

Supplement: Supplementary Figure 2 — Relative abundance of Prevotella sp. within severity groups. Class 1: Severity group 1; Class 2: Severity group 2; Class 3 Severity group 3. The data was obtained using QIIME2 together with LEfSe (Linear discriminant analysis Effect Size). [file Image_2.tif]

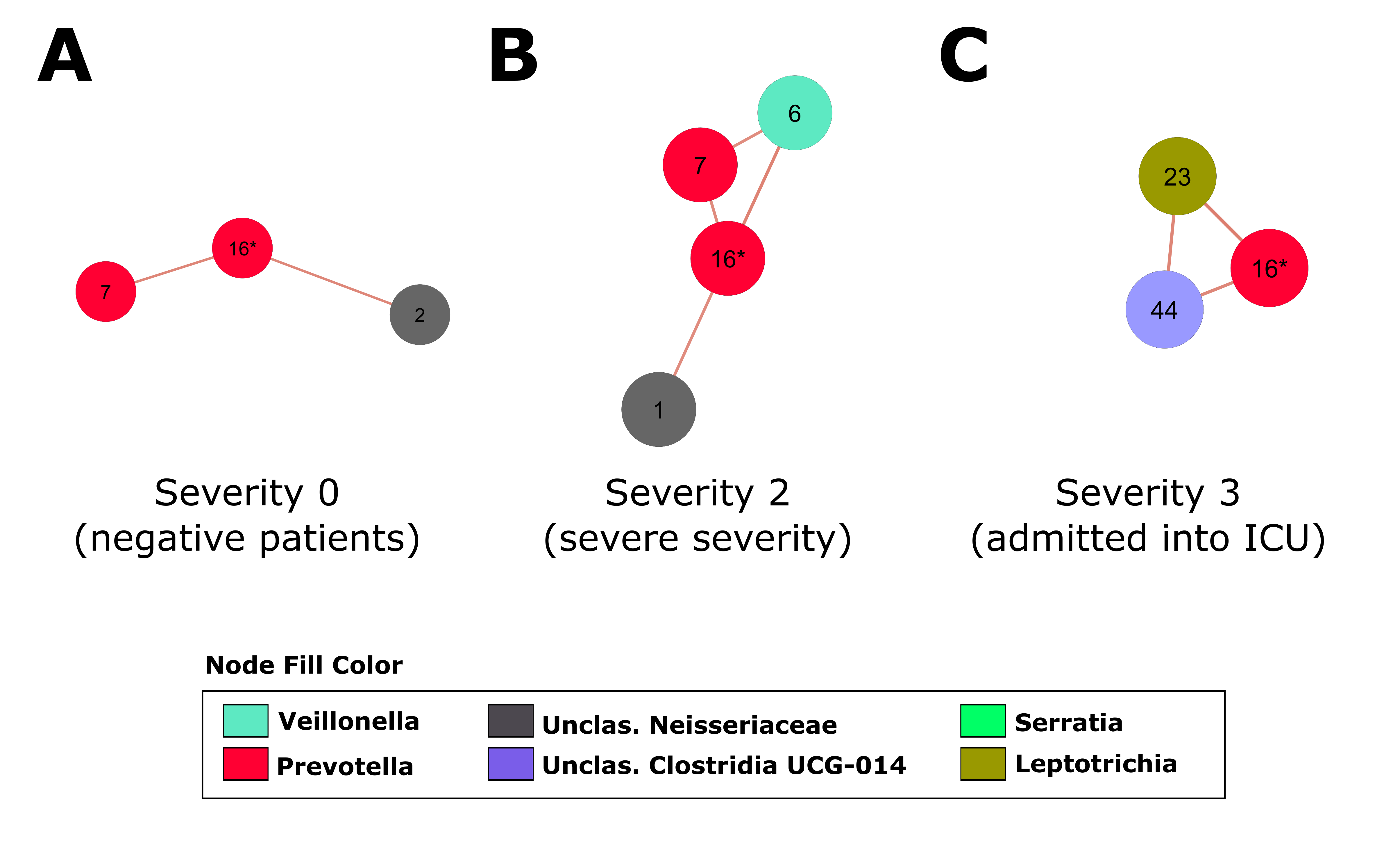

Supplement: Supplementary Figure 3 — Co-abundance network showing only first-degree neighbors of OTU 16 (Prevotella sp.). (A) Severity group 0, (B) Severity group 2, and (C) Severity group 3. [file Image_3.tif]
